# Research Trend Visualization by MeSH Terms from PubMed

**DOI:** 10.3390/ijerph15061113

**Published:** 2018-05-30

**Authors:** Heyoung Yang, Hyuck Jai Lee

**Affiliations:** Kore Institute of Science and Technology Information, 66, Hoegi-ro, Dongdaemun-gu, Seoul 02456, Korea; hlee@kisti.re.kr

**Keywords:** PubMed, medical subject headings, keyword network, MeSH correlations, MeSH Net

## Abstract

*Motivation*: PubMed is a primary source of biomedical information comprising search tool function and the biomedical literature from MEDLINE which is the US National Library of Medicine premier bibliographic database, life science journals and online books. Complimentary tools to PubMed have been developed to help the users search for literature and acquire knowledge. However, these tools are insufficient to overcome the difficulties of the users due to the proliferation of biomedical literature. A new method is needed for searching the knowledge in biomedical field. *Methods*: A new method is proposed in this study for visualizing the recent research trends based on the retrieved documents corresponding to a search query given by the user. The Medical Subject Headings (MeSH) are used as the primary analytical element. MeSH terms are extracted from the literature and the correlations between them are calculated. A MeSH network, called MeSH Net, is generated as the final result based on the Pathfinder Network algorithm. *Results*: A case study for the verification of proposed method was carried out on a research area defined by the search query (immunotherapy and cancer and “tumor microenvironment”). The MeSH Net generated by the method is in good agreement with the actual research activities in the research area (immunotherapy). *Conclusion*: A prototype application generating MeSH Net was developed. The application, which could be used as a “guide map for travelers”, allows the users to quickly and easily acquire the knowledge of research trends. Combination of PubMed and MeSH Net is expected to be an effective complementary system for the researchers in biomedical field experiencing difficulties with search and information analysis.

## 1. Introduction

The size of the literature in biomedical domain grows exponentially [1]. Some of the reasons for the growth are the ease of the Internet access, breakdown of interdisciplinary boundaries due to genome-scale instruments, and increasingly interdisciplinary nature of research and development [1,2,3]. Professionals such as researchers and educators experience difficulties keeping abreast of the literature in their research areas because of the massive amount of literature [4].

PubMed, provided by the National Center for Biotechnology Information (NCBI), has served as the primary information source based on search tool and the literature in biomedical domain. As of 2017, PubMed comprises more than 27 million publications in the broad and up-to-date sources such as biomedical literature from MEDLINE, life science journals, and online books according to the website (https://www.ncbi.nlm.nih.gov/pubmed/). The total number of publications indexed by PubMed is continuously on the rise; the annual average growth rate was reported to be 4% [2]. Owing to the ever-growing size of the literature in PubMed, users often confront with long lists of search results, which make it challenging to find the information and knowledge they want.

To overcome this difficulty, PubMed complementary tools, often called “PubMed derivatives”, have been developed. Lu (2011) [2] and NCBI (2012) [5] surveyed “PubMed derivatives” and typified the characteristics of each. They showed 37 tools and categorized those into five groups: ranking search results, clustering results into topics, extracting and displaying semantics and relations, improving search interface and retrieval experience [2,5]. Most of these tools provide search results in the format of “publication list”. A few give the result in “graph” or “wordcloud”. Besides the tools mentioned above, other tools were also developed: MeSHmap [3], MeSHy [6] and Meshable [7]. These give the search result in the list of keywords and the list of keyword categories, the list of keyword pairs, and keyword list interacting with search results pages, respectively.

The development history of PubMed derivatives spans from 2001 to 2016. A long history of development reflects various user needs on the one hand. On the other hand, it could also mean that there has not been a “killer application” as if Google made many services extinct since its appearance in the web search market. Another implication can be found from the current operability of the tools. We checked the current status of the aforementioned tools, only 18 of them were found to be currently operable considering some of the commercial services operable by the existence of their website. Maintenance of the tools is another important issue regarding the validity of tools. The last update of the 18 operable tools goes back several years, which indicates that the maintenance is not supported by the developers anymore. The links to their websites provided by NCBI seem to be broken and the latest information on them cannot be found on the Internet.

The current status of the PubMed derivatives reflects that they did not gain much popularity from the users; PubMed users do not seem to be fully satisfied with these derivatives in other words. The reason for this dissatisfaction could be attributed to two facts: the format and insufficient utility of the output. Many of the PubMed derivatives give the output in the format of “publication list”. When a user is given a long list of publications from the tool, the user will have to use a line-by-line approach to find a specific publication or to extract knowledge from the list depending on the purpose of the search. Even though the tools use different ranking algorithms, and hence the order of the articles in the list is different, from that of PubMed, the user’s task remains basically the same. It should also be noted that the utility of the tools’ outputs may not be sufficient. Even though many of the tools provide statistical analysis and clustering (or grouping) results in addition to the simple list, they may not be able to give the users much more benefits than PubMed because PubMed also provides analytical features such as yearly statistics with several kinds of filters. Hence, the users would not choose the “new tools” at the expense of the effort learning how to use them unless they provide more benefits than the already accustomed tool, PubMed, does.

The very basic function of PubMed is to provide the list of publications corresponding to a user’s search query defining a research area (or a research topic). The user reviews the list in a line-by-line approach to acquire the knowledge structure of the research area. The overall knowledge structure of the research area is built by combining every bit of knowledge. This is often referred to as a cognitive process. If the search result is given as a science map, which is one of the information visualization techniques intended to provide a geography of a research area so that the search and retrieval of desired information out of large collection of information can be carried out via a user-friendly way, an intuitive process so to speak, it could be a good PubMed derivative. [8]. A good PubMed derivative is also required to selectively deliver information on emerging research areas needed by researchers. This requires the removal of information that is not part of the emerging research area, i.e., noise removal.

We have studied how to generate essence of knowledge structure by applying Social Network Analysis (SNA) to PubMed literature. SNA started from sociometric analysis and Graph Theory, and was applied to a wide range of social phenomena, psychology, and economics, and recently its use has expanded to include research into the characteristics very complex networks in neurobiology, statistical physics, etc. [9,10,11]. Otte and Rousseau (2002) [12] stated that SNA is more a strategy to provide a viewpoint to explore the structure of social networks, rather than a methodology used in a specific field, and explained some examples in the information science. SNA is applied to not only the analysis of relationship between people, institutions and journals, but also the analysis of relationship between keywords [13,14]. We noted the relationship of medical keywords in the PubMed literature. To understand the latest research trends, it is necessary to analyze which medical keywords are emerging, but the keyword alone is not enough to explain the research topic. Knowing how the keywords are related to each other and how they are connected helps better understand the research topic. We studied a methodology to visualize a social network composed of emerging medical keywords in PubMed literature, and introduced an application using this method.

A prototype application is proposed in this paper as a new PubMed derivative including a new method for research trend visualization of a specific research topic. The application starts in quite a similar way to the conventional PubMed search. The user simply enters a search query, and then the application retrieves corresponding publications from PubMed using the Entrez Programming Utility, an API provided by NCBI. Based on the bibliography of the publications, the research trend is visualized by the method proposed in this study. In the following sections of this article, a review is given on selected PubMed derivatives providing the output in a format other than “publication list”, that is, a graph of keyword relation, etc., to help the users acquire the knowledge structure of a specified research area. Then, the research visualization process proposed is given. A research area defined by the search query (immunotherapy and cancer and “tumor microenvironment”) is applied to our process as a case study. The query is a simple sequence of typical keywords in the research area, which is composed without any “tuning” by the field experts or analysts. In the final section of this article, the implication, possible applications, limitations of the proposed process and the future plan are mentioned.

## 2. Review for Selected PubMed Derivatives

### 2.1. GoPubMed 

GoPubMed [15] utilizes Gene Ontology (GO) terms, which are structured, controlled vocabularies and classifications for the annotation of genes, gene products and sequences [16]. GO terms are comprised of over 19,000 terms organized in three sub-ontologies for cellular location, molecular function and biological process. GoPubMed extracts GO terms from the abstracts of the publications in PubMed search result, and groups the publications according to the GO terms. The users are given the PubMed search result as a list in which the publications are categorized according to the GO terms. Such a grouping can provide the users an easy way to identify publications with research theme (or concept), and the search can be refined using the sub-theme. The original version of GoPubMed provided the hierarchy of GO terms, which is not available in the current version. Instead, the current version provides Medical Subject Heading (MeSH) terms together with the GO terms. These terms are referred to as “concepts” in GoPubMed, where the details are available at http://help.gopubmed.com/. The advantage of GoPubMed is that the users can overview the search result more easily by using the “concepts”. However, GoPubMed provides the concepts as a list without giving any relationship between them, and hence the users have to go line-by-line to find the knowledge structure.

### 2.2. Semantic MEDLINE

Semantic MEDLINE [17] is a web application using two existing tools: SemRep [18] based on Unified Medical Language System (UMLS) [19] and automatic summarizer [20]. SemRep is a general knowledge-based semantic interpreter, based on Unified Medical Language System (UMLS). Semantic MEDLINE extracts predications based on UMLS concepts from the publications in the PubMed search result. The list of predications is entered into the automatic summarizer and then assorted to the list of semantic condensate (list of UMLS concepts), which is provided to the users. In other words, each publication in the PubMed search result is matched with UMLS concept. This matching is carried out in sentence-based way, and hence multiple UMLS concepts are matched to a single publication. Semantic MEDLINE, as a result, gives lists of multiple interlinked UMLS concepts per publication. When the user clicks one UMLS concept, Semantic UMLS shows the elements that comprises the UMLS and their relationships in graphical way. The user can understand the knowledge structure from the UMLS concept and can find the publication. The advantage of Semantic MEDLINE is the easy literature search and knowledge exploration by the summarization of semantic condensate of biomedical information. Because the SemRep process, extracting the UMLS concepts from the publications, is a slow process, it is carried out in off-line and the predications extracted are stored in a separate database. This could yield the problem such that the result may not be up-to-date and the knowledge structure not pre-defined in UMLS cannot be found.

### 2.3. MeSHy 

Most of the previous PubMed derivatives deal with the occurrence (or simple count)-based statistical information. Theodosiou et al. pointed that the tools for discovering unusual and unanticipated information have never been developed, and proposed MeSHy based on the statistical characteristics of co-occurrence of MeSH term pairs [6]. MeSHy calculates the score of MeSH term pairs, which is a kind of relationship between the probabilities of MeSH term pairs. Each of the MeSH terms is extracted from the publications in the PubMed search result and its probability within the body of the search result is calculated from its occurrence count. Comparing the score of MeSH term pairs with the probability of co-occurrence MeSH term pairs could provide unanticipated MeSH term pairs in statistical point of view. MeSHy then provides the list of MeSH term pairs sorted by the score accompanied by popularity (or rareness) of each MeSH. Links to the publications where each of the MeSH term pair occurs are provided as well. MeSHy is specifically designed to provide the unanticipated knowledge domains that might have implications to the users so that they can explore the novel and promising research areas. Even though this does not fit the purpose of looking at the whole when general researchers explore the domain of knowledge, this can be helpful in terms of finding research ideas that come to the researchers as serendipity.

## 3. Methodology

This study aimed to propose a new method for generating the most recent knowledge structure of a research area which is defined by a user’s search query to PubMed. The output format is to be in a *visualized manner* not a simple list of search result, more specifically a network graph consisting of biomedical terms, because the former is a more appropriate way in assisting the users with exploring the knowledge structure *intuitively*. Providing the most up-to-date information is crucial, and hence the method will gather bibliographic information from PubMed every time the user specifies a search query. The primary biomedical terms to be analyzed are MeSH, and the reason for selecting MeSH is given in the following section together with the detailed process on preparing the dataset and generating the MeSH network.

### 3.1. About MeSH

Medical Subject Headings (MeSH) is a controlled vocabulary thesaurus provided by US National Library of Medicine (NLM). MeSH consists of sets of biomedical terms in a hierarchical structure helping the literature search at various levels of biomedical domains. According to the 2017 MeSH data file, which is available at NLM’s web page (https://www.nlm.nih.gov/mesh/filelist.html), MeSH consists of about 57,800 biomedical terms for MeSH Descriptors (Main Headings) and 82 Qualifiers (Subheadings), where the former is used to index publications in MEDLINE for topical headings and the latter to confine the subject to a particular aspect of Main Headings. Both terms are searchable in PubMed, and updated by NLM on an annual basis. In general, the publication indexed by PubMed contains several lines of MeSH terms, each of which is in the format of combining one Main Heading and one or multiple Qualifiers. MeSH can be used without additional cleansing, while the conventional keywords, such as author keywords, requires massive amount of cleansing for proper usage. For these reasons, MeSH is a valuable material for identifying research trend from biomedical publications, and used as the primary biomedical terms for the method proposed in this study.

### 3.2. MeSH Dataset

**Figure 1:**
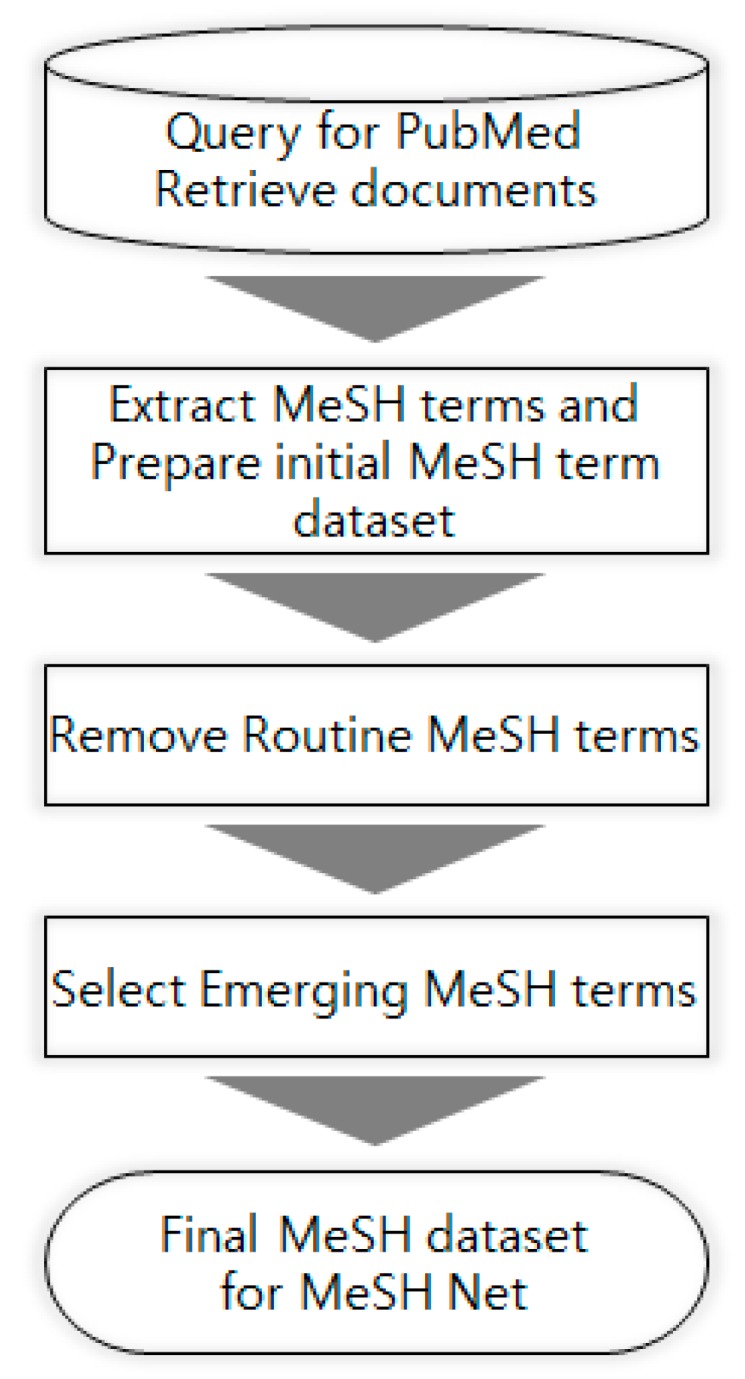
Flowchart for MeSH term dataset preparation.

Every publication indexed in the MEDLINE database which is primary component of PubMed literature has at least one, very often multiple, MeSH terms. Collecting all the MeSH terms in a set of publications corresponding to a search query to PubMed may generate “information overload” depending on the size of the set. Therefore, selecting only the MeSH terms which are considered “noteworthy” is a key to successful analysis and identifying the knowledge structure in them. The application proposed in this study includes several steps for this purpose, as shown in Figure 1. Since this application focuses on the most recent knowledge structure of a research field, the final MeSH terms are collected from the publications of the most recent three years (time period is an adjustable variable in the application). The following section gives the detailed information on each of the steps mentioned above.

#### 3.2.1. PubMed Search and Initial MeSH Terms

Using the Entrez Programming Utility provided by NCBI, a search query (immunotherapy and cancer and “tumor microenvironment”) is submitted to PubMed and corresponding publications are collected for the case study on the research area. Preparation of the initial MeSH terms starts from defining the *lifetime* of the research area using the annual publication count. The most recent calendar year in the publication set is set to Y_last_ while the rolling year can also be used alternatively to emphasize the recent publications in the set. Every three-year period starting from Y_last_ backwards is grouped to form Time Periods, and only those with the annual average publication count greater than 10 are selected, which is because we think that the average publication count should be at least 10, indicating that research has begun in the field. Each of the Time Periods is given as *T_1_*, … *T_N_*, where N is the total count of the Time Periods. All the MeSH terms are extracted from each of Time Periods and they are referred to as the *initial* MeSH terms. The occurrence count of *initial* MeSH term M_i_ is measured for each of Time Periods, and given as $C{\left(M_{i}\right)}_{T_{1}}$, $C{\left(M_{i}\right)}_{T_{2}}$, … $C{\left(M_{i}\right)}_{T_{N}}$.

#### 3.2.2. Removal of Routine MeSH Terms

Some of the MeSH terms must be removed from the initial MeSH term set to prepare the “noteworthy” MeSH terms. The first type of MeSH terms to be considered is the “Routine MeSH terms” which appear dominantly throughout the lifetime of the research area because it is so broad and routine. The term “Cancer” is a good example of the Routine MeSH term in the research field “Targeted Therapy for Cancer”. Most publications in this area contain the term Cancer, which cannot attract the professionals in this area. The professionals would be more interested in new emerging terms with small frequencies rather than Routine MeSH terms with large frequencies. Moreover, it could bring a serious bias, acting as a “strong hub”, to the MeSH term network, especially when the correlations between the terms are measured on occurrence count basis (see Section 3.2.3 for detail). The impact of the strong hub to a network is so great that the other characteristics of the network may become relatively small and difficult to be observed. Thus, for any MeSH term $M_{i}$ in the initial MeSH term set, if the occurrence counts as $C{\left(M_{i}\right)}_{T_{1}}$, $C{\left(M_{i}\right)}_{T_{2}}$, … $C{\left(M_{i}\right)}_{T_{N}}$ for all of the time period 1, 2, … N are positive, it is considered as the Routine MeSH term and removed from the dataset.

#### 3.2.3. Selection of Emerging MeSH Terms

Growth pattern is another aspect of MeSH terms to be considered to prepare the “noteworthy” MeSH terms. Users would be interested in the recent terms, and they would be most interested in the recent terms of increasing occurrence counts. Therefore, the method proposed in this study focuses on “Emerging MeSH terms”. By doing so, the users would be able to identify the knowledge structure of an emerging area more intuitively, otherwise they would have to deal with overall status of the research area. The Emerging MeSH terms are defined as the MeSH terms that have positive MeSH term count increment in the Time Period *T_N_* from the previous *T_N−1_*, which is written as $C{\left(M_{i}\right)}_{T_{N}}-C{\left(M_{i}\right)}_{T_{N-1}}>0$ and $C{\left(M_{i}\right)}_{T_{N}}\geq 3$ for the condition of average count at least once a year.

### 3.3. Measurement of the MeSH Term Correlations

Two MeSH terms comprising a pair are said to co-occur in a set of publications when there is at least one publication containing both. Based on the principle of co-word analysis, the strength of their relationship is interpreted [21]; the more these two words (or terms) co-occur, the stronger the relationship between them is. In a conventional way, the correlation is measured based on the co-occurrence count of the terms. A correlation matrix is given by repeating this measurement over all of the pairs in the dataset and presenting the result in the format of matrix. Then, a network structure is generated based on the correlation matrix.

The structure of the network, however, could be seriously biased by the dominating terms, the Routine MeSH Terms for instance, in the term list when the correlation matrix is measured based on the conventional way. Beside the Routine MeSH terms, other terms such as “Trendy Terms” and “Query Terms” could also bias the network in the same way as the routine terms do. The Trendy Terms, which are very popular, and thus frequently used by the researchers in the research area in a given time period, may appear in a large portion of the publications creating many connections to other terms and acting as the “strong hub”. Query Terms, the terms used in a search query, are another type of the candidate for the strong hub. Although the Query Terms may not “literally” be expressed as MeSH term, there is a high possibility that they could be found in the MeSH term inventory if they are biomedical terms. In such a case, they will be included in the network acting as the strong hub.

To remedy these problems, an alternative method to prepare the correlation matrix is proposed in this study; the similarities of the titles of the publications containing the MeSH terms in a pair is used instead of co-occurrence count of the terms. The titles are the essence of the research topic enclosed in the publications, and generally the most recent terms in the research area are selected. The title similarities have nothing to do with the co-occurrence count of MeSH terms, and thus the bias by the dominant terms aforementioned, such as the Routine, Trendy and Query terms, acting as the strong hubs can be avoided. The following shows how the title similarities are used to measure the correlation between the MeSH terms.

Title Keywords (TK) (both words and noun phrases) are extracted from the titles of the publications by applying Natural Language Processing (NLP). A bibliometric data management tool, the VantagePoint provided by the Search Technology, Inc. (www.thevantagepoint.com), is used to apply the text mining processes: NLP, extracting and cleansing the keywords. For the time period $T_{N}$, MeSH-TK co-occurrence matrix is generated as shown in Table 1. For the two MeSH terms $M_{i}$ and $M_{j}$, the vector $\vec{V\left(M_{i}\right)}$, $\vec{V\left(M_{j}\right)}$, and cosine similarity $\mathrm{Sim}\left(M_{i},\;M_{j}\right)$ are given as follows:

(1) $$\vec{V\left(M_{i}\right)}=vector\left(C\left(TK_{1}|M_{i}\right),\;C\left(TK_{2}|M_{i}\right),\;C\left(TK_{3}|M_{i}\right),\;\ldots ,\;C\left(TK_{K}|M_{i}\right)\right)$$

(2) $$\vec{V\left(M_{j}\right)}=vector\left(C\left(TK_{1}|M_{j}\right),\;C\left(TK_{2}|M_{j}\right),\;C\left(TK_{3}|M_{j}\right),\;\ldots ,\;C\left(TK_{K}|M_{j}\right)\right)$$

(3) $$\mathrm{Sim}\left(M_{i},\;M_{j}\right)=\;\frac{\vec{V\left(M_{i}\right)}\;\cdot \;\vec{V\left(M_{j}\right)}}{\left|\vec{V\left(M_{i}\right)}\right|\;\;\left|\vec{V\left(M_{j}\right)}\right|},$$

where $C(TK_{k}|M_{i})$ is the occurrence count of the title keyword $TK_{k}$ in the publication set to which the MeSH term $M_{i}$ is assigned. *K* is the number of title keywords extracted by text mining on the whole literature set retrieved from PubMed by the search query.

### 3.4. MeSH Net by Pathfinder Network Algorithm

**Figure 2:**
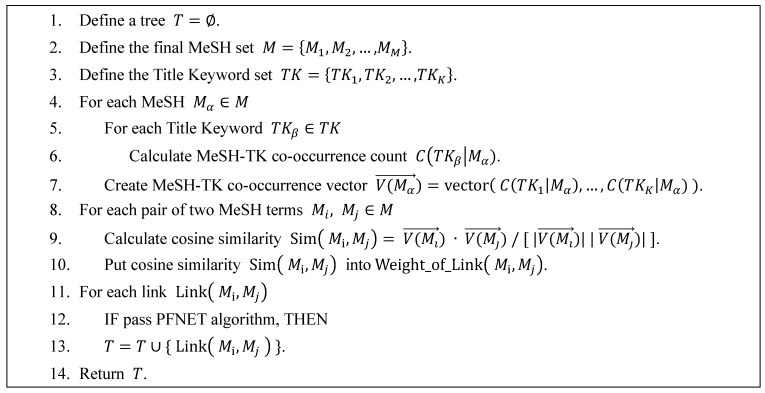
The pseudo-code for measurement of the Medical Subject Headings (MeSH) term correlations and the adoption of Pathfinder Network (PFNET) algorithm.

When a network contains many nodes and links to be depicted, a “complex network” so to speak, it often becomes over-crowded leaving the readers hindered from identifying the salient structure of the network. A practical solution is to reduce the number of links in the network. There are several algorithms developed for the link reduction. The key issue is whether the algorithm preserves the underlying topological properties. The link reduction algorithm used in this study is a Pathfinder Network (PFNET) [22,23], which is one of the two most popular link reduction algorithms in information visualization together with the Minimum Spanning Tree (MST). Some studies suggest that PFNET, which is originally developed to depict the salient network consisting of “concepts”, is more suited to knowledge structuring [24,25,26] because MST has the potential to eliminate links that may be significant. Eliminating potentially significant links can lower the accuracy of knowledge structuring. PFNET (PFNET has two parameters, q and r. PFNET (r = ∞, q = n − 1) is the minimal PFNET which has the fewest number of links among all possible PFNETs. PFNET (r = ∞, q = n − 1) also include all links that are in any MST networks) includes all of potentially significant links, and hence is a better way for effective link reduction without losing important information. Several studies have used to explore the structure of knowledge, research trends or research fronts using the PFNET [27,28,29,30,31]. The PFNET algorithm is applied to the MeSH term correlation matrix obtained from both the conventional way and the one proposed in this study for comparison. Figure 2 shows the pseudo-code for measurement of the MeSH term correlations and the adoption of PFNET algorithm.

## 4. Results and Discussion

As mentioned in Section 3.2.1, a search query (immunotherapy and cancer and “tumor microenvironment”) was submitted to PubMed, and the bibliographic information on 1935 publications returned was collected. This publication set was treated as the research area for the case study of the method proposed in this study. The time span of the research area is 1993–2017. Complete calendar year was used to build the Time Periods, and hence Y_last_ was set to 2016. The second column in Table 2 shows the annual publication count based on which the lifetime of the research area is measured. We repeat the Time Period composition every three years from Y_last_, 2016, to the past, e.g., 2016–2014, 2013–2011, …, 1995–1993. In the Time Periods 2016–2014, 2013–2011, 2010–2008, 2007–2005, and 2004–2002, the average publication count is greater than 10 according to the third column of Table 2. Although there were publications from 1993, the annual publication count does not exceed 10 in the time periods of 2001–1999, 1998–1996, and 1995–1993. Therefore, five periods were found to satisfy the criterion for the Time Period, i.e., average annual publication count greater than 10, resulting in the lifetime of 15 years for the given research area. Then, the time period of 2004–2002 was set as Time Period 1 (*T_1_*) for the starting point of this research field, and 2007–2005, 2010–2008, 2013–2011, and 2016–2014 were set as Time Periods 2–5, respectively. The number of the initial MeSH terms extracted from the lifetime range of Time Periods 1–5 is 1758 in total.

The Routine MeSH terms, defined in Section 3.2.2 as the MeSH terms appearing in all of the Time Periods, were removed from the initial MeSH term set, resulting 1644 terms. After removing the Routine MeSH terms, the Emerging MeSH terms, defined in Section 3.2.3 as the MeSH terms with the occurrence count in *T_N_* greater than *T_N-1_* and greater than or equal to 3 (in this case $T_{5}>T_{4}$ and $T_{5}\geq 3$) were selected and used as the final MeSH terms for the case study. By following these processes, the number of the MeSH terms was reduced to 266, 15.1% of the initial MeSH terms.

**Figure 3:**
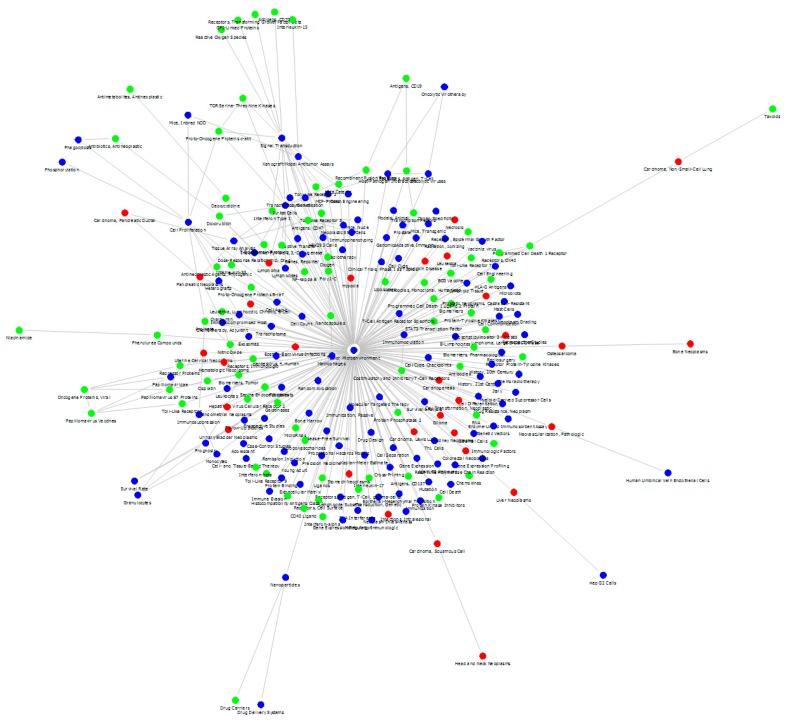
Medical Subject Headings (MeSH) network based on the MeSH term correlation matrix prepared from the MeSH term co-occurrence count for the case study research area. The colors of the nodes represent different characteristics of MeSH terms: red dots for Disease, green for Chemicals and Drugs, and blue for other MeSH terms.

Figure 3 is a MeSH PFNET network based on the MeSH term correlation matrix prepared from the MeSH term co-occurrence count. Figure 3 is illustrated through the Fruchterman and Reingold algorithm (one of the network visualization algorithms [32]) using the SNA software Netminer, by Cyram, Inc, (www.cyram.com). This network has a structure that the term “tumor microenvironment”, which is located in the center of the graph, acts as a strong hub. This term is a part of the search query, and thus classified as a Query Term. At the same time, it is a Trendy Term according to the experts in the research field. In such a network (Figure 3), it is very difficult to capture the salient knowledge structure because the impact of the strong hub is so great that the remaining part of the connections in the network is buried underneath. This term is directly connected to 74.4% of the terms in the network (198 out of 266 MeSH terms).

**Figure 4:**
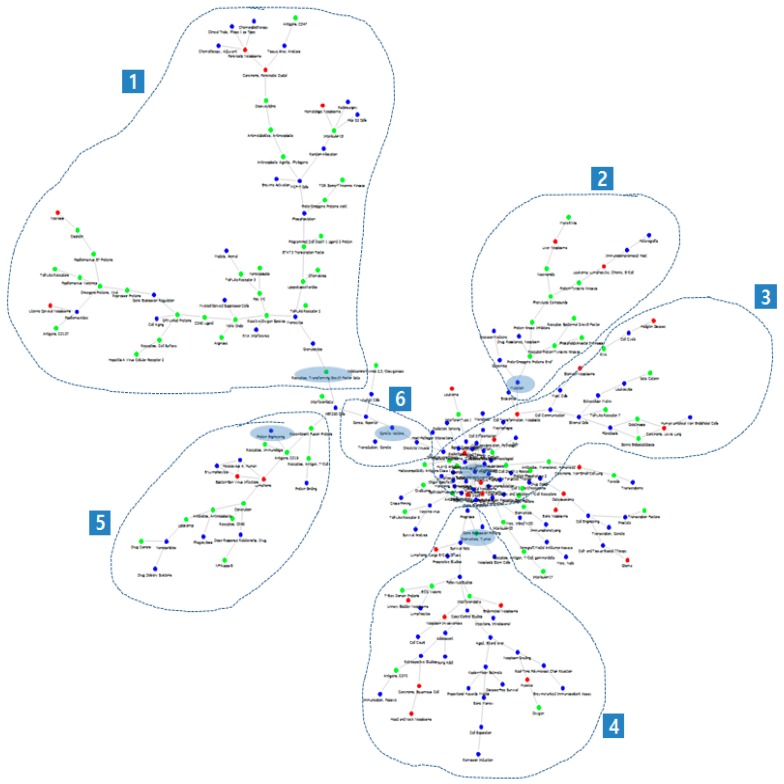
Medical Subject Headings (MeSH) network based on the MeSH term correlation matrix prepared from the title similarities for the case study research area. This network is referred to as “the MeSH Net”. The six sub-areas in dotted lines are in good agreement with the actual research activities in as confirmed by an expert in the corresponding research area. The representative MeSH terms for each sub-area are shown marked with blue shades. The colors of the nodes are the same as those in Figure 3.

Figure 4 is a MeSH PFNET network based on the MeSH term correlation matrix prepared from the title similarities, which is the alternative method proposed in this study. Figure 4 is also illustrated through the Fruchterman and Reingold algorithm. The prototype application developed in this study provides a zooming function so that the users can identify the connection details of the network when necessary. The colors of the nodes are given according to the nature of the corresponding MeSH terms: red for Disease, green for Chemicals and Drugs, and blue for others. This coloring can help the readers better understand the characteristics of the relationships between the MeSH terms: target diseases for chemicals and drugs, for example.

Quite contrary to the network in Figure 3, it has a well-defined structure consisting of branches, which can be interpreted as the sub-areas (research topics comprising the research field). Sub-branches of a large branch can be considered the subsidiary topics. This is a feature of the PFNET algorithm: even without clustering or community detection, branches are formed by research topics, so it is possible to grasp detailed research topics just by looking at the network graph. The validation of Figure 4 was carried out by the experts in this research area, who gave a clear definition on the six sub-areas by grouping the MeSH terms in the network and assigning a representative MeSH term per sub-area: Receptors, Transforming Growth Factor beta; Mutation; Tumor microenvironment; Biomarkers, Tumor; Protein Engineering; and Genetic vectors. These terms are marked with blue shades and underscore in Figure 4 and Table 3 which shows some of major MeSH terms in each group. They also confirmed that the network structure (the sub-areas and the subsidiary topics) was in good agreement with the actual activities in the recent research area. The network in Figure 4 is referred to as the “MeSH Net”.

What is remarkable with this Map is that it was created only with the search query and statistical processes without the help or intervention of the experts in the research area. The search query was a very simple list of typical keywords in the research area, not the one with a complex search expression that professional analysts often make. When using this method, therefore, the user only needs to input a search query for the research area to explore and wait until the calculation is finished. The user will be given a network in a graphic mode with the essential information on the research trend and the knowledge structure which are very intuitive for understanding the result. Common search tools, including PubMed, provide search results in a publication list. It the number of searched publications is 1000 and a list of 10 publications is displayed per search page, the user needs to click on up to 100 pages to check the titles of all publications. The method we introduce is to structure the knowledge of 1000 publications using MeSH terms and provide them as one MeSH Net graph which could help users to understand intuitively the recent research trends.

## 5. Conclusions

The increase of publications in biomedical field makes the researchers and educators experience difficulties with keeping track of the literature in their research areas. Various PubMed derivatives have been developed to assist them as the complementary tools to PubMed. Most of these tools, however, including PubMed itself, give the output as the list of search result, which requires much time and effort from the users either to locate a publication or to extract knowledge from it. Although some PubMed derivatives use different ranking algorithms from PubMed, the users still need to collect bits of knowledge to build the overall knowledge structure; a cognitive way, so to speak. An alternative way would reduce the amount of time and effort by providing an easier way to explore the knowledge structure of a research area is needed; an intuitive (or graphical) way.

What is proposed in this paper is a prototype application with an alternative method to explore the research trend of a research area using a network graph. We have studied a methodology to visualize a social network composed of emerging medical keywords in PubMed literature, so-called MeSH terms, a controlled biomedical vocabulary thesaurus. The new method includes the mechanism to select only the “noteworthy” MeSH terms from those extracted from the publications in a research area defined by a search query, a simple list of keywords, to PubMed. Another unique feature of the method is that the network graph is generated via the similarities of the titles of the publications, which is different from the conventional method using the co-occurrence count of the terms. According to a case study, it was found that the resulting network graph is not biased by the dominating terms such as Routine, Trendy and Query terms, and that the knowledge structure obtained showed the structure made of six well-defined branches. The experts in the corresponding research area confirmed that the MeSH terms selected and the network structure obtained were in good agreement with the actual activities in the research area. The whole analytical process does not need any knowledge of experts once the research area to be analyzed is given by the user, which implies that the application can act as an automated technology intelligence tool.

The prototype application that implements the method for generating MeSH Net is believed to be helpful for the users to explore the research trend of their research areas of interest. The prototype application would have some additional functions to improve performance. One of the examples is the “Click and Link”, which opens a pop-up window containing the list of the publication of PubMed including the MeSH term when a MeSH term in the network graph is clicked by the user. It is also possible to link to other literature databases including MeSH terms, ClinicalTrials.gov database for example. The link to a list of clinical trial studies including the MeSH term would be added as well. One concern with the operation of the application is the running speed. If the search query entered by users is too broad and covers many documents, it could take a considerable amount of time to download the document information and calculate similarities to draw the network. A further study will be carried out on the speed problem according to the number of documents and to scale the size of the analytical literature to maintain the speed that the users can accept. Overall, the application for the MeSH Net with the aforementioned features is expected to provide users an easier way to explore the research areas of their interests.

## Figures and Tables

**Table 1:** MeSH-TK co-occurrence matrix for a time period $T_{N}$.

TK MeSH	$TK_{1}$	$TK_{2}$	$TK_{3}$	…	$TK_{K}$
$M_{1}$	$C(TK_{1}|M_{1})$	$C(TK_{2}|M_{1})$	$C(TK_{3}|M_{1})$	…	$C(TK_{K}|M_{1})$
$M_{2}$	$C(TK_{1}|M_{2})$	$C(TK_{2}|M_{2})$	$C(TK_{2}|M_{2})$		$C(TK_{K}|M_{2})$
$M_{3}$	$C(TK_{1}|M_{3})$	$C(TK_{2}|M_{3})$	$C(TK_{3}|M_{3})$		$C(TK_{K}|M_{3})$
…	…	…	…	…	…
$M_{M}$	$C(TK_{1}|M_{M})$	$C(TK_{2}|M_{M})$	$C(TK_{3}|M_{M})$		$C(TK_{3}|M_{M})$

**Table 2:** Measuring the lifetime of a research area for the case study.

Year (Y)	Publication Count	Average Publication Count	Time Period (T)
2017	186	NA	NA
2016	395	318.3	5
2015	325		
2014	235		
2013	197	156.7	4
2012	163		
2011	110		
2010	68	53.7	3
2009	48		
2008	45		
2007	54	31.7	2
2006	27		
2005	14		
2004	12	11.3	1
2003	14		
2002	8		
2001	4	4.3 *	*
2000	4		
1999	5		
1998	10	*	
1997	0		
1996	1		
1995	2	*	
1994	5		
1993	3		

* Not eligible for Time Periods.

**Table 3:** Examples of major Medical Subject Headings (MeSH) terms in each group of Figure 4.

Major MeSH Terms	
**in Group 1**	**in Group 2**
Receptors, Transforming Growth Factor beta	Mutation
Oncogene Proteins, Viral	Proto-Oncogene Proteins B-raf
Nitric Oxide	Phenylurea Compounds
Poly I-C	
GPI-Linked Proteins	
MCF-7 Cells	
Pancreatic Neoplasms	
**in Group 3**	**in Group 4**
Tumor Microenvironment	Biomarkers, Tumor
Immunomodulation	Survival Rate
Cell Transformation, Neoplastic	Follow-Up Studies
Serine Endopeptidases	Kaplan-Meier Estimate
Gelatinases	BCG Vaccine
Molecular Targeted Therapy	
**in Group 5**	**in Group 6**
Protein Engineering	Genetic Vectors
Herpesvirus 4, Human	Oncolytic Viruses
Epstein-Barr Virus Infections	

Underscored Medical Subject Headings (MeSH) terms are the representative terms for corresponding groups.
